# Gait speed-dependent modulation of paretic versus non-paretic propulsion in persons with chronic stroke

**DOI:** 10.1186/s12984-025-01620-0

**Published:** 2025-05-08

**Authors:** Joost Biere, Brenda E. Groen, Carmen J. Ensink, Jorik Nonnekes, Noël L. W. Keijsers

**Affiliations:** 1 Department of Research, Sint Maartenskliniek, PO Box 9011, 6500 GM Nijmegen, The Netherlands; 2 Department of Sensorimotor Neuroscience, Donders Institute for Brain, Cognition and Behaviour, Radboud University, Nijmegen, The Netherlands; 3 Donders Institute for Brain, Department of Rehabilitation, Cognition and Behaviour, Radboud University Medical Centre, Nijmegen, The Netherlands; 4 Centre of Expertise for Parkinson & Movement Disorders, Radboud University Medical Centre, Nijmegen, The Netherlands; 5 Department of Rehabilitation, Sint Maartenskliniek, Nijmegen, The Netherlands

**Keywords:** Gait, Stroke, Propulsion, Gait speed, Symmetry, Hemiparesis, Rehabilitation

## Abstract

**Background:**

Persons with chronic stroke (PwCS) exhibit impaired paretic propulsion generation. Consequently, PwCS walk slower than healthy peers and rely more on their non-paretic leg, leading to propulsion asymmetry. However, it remains unclear how propulsion symmetry is influenced by walking at various gait speeds. This study aimed to investigate the relation between gait speed and propulsion symmetry in PwCS and controls.

**Methods:**

Fifteen PwCS and sixteen healthy controls walked on an instrumented treadmill at randomized speeds, ranging from 0.2 m/s to comfortable walking speeds for PwCS or 0.4 to 1.6 m/s for controls, with 0.2 m/s increments. PwCS continued to their maximum speed with 0.1 m/s increments. Propulsion, derived from the anteroposterior component of the ground reaction force, was defined as propulsion peak and propulsion impulse. The primary outcome was propulsion peak and impulse symmetry (paretic propulsion / total propulsion), with secondary outcomes being propulsion peak and impulse per leg. The relationship between gait speed and propulsion metrics was analyzed using linear mixed models (LMM).

**Results:**

PwCS exhibited clear propulsion peak and impulse asymmetry across all gait speeds, while controls maintained symmetrical propulsion. LMMs revealed no change in propulsion peak symmetry with gait speed (β = 0.12, SE = 0.090, p = 0.19), with considerable variability among PwCS. Propulsion impulse symmetry improved with increasing gait speed (β = 0.39, SE = 0.048, p < 0.001), especially in PwCS who had greater asymmetry at comfortable walking speed. Propulsion peak and impulse increased with gait speed in both legs for PwCS and controls. The propulsion peak increase was stronger in the non-paretic compared to the paretic leg (0.16 ± 0.043 vs. 0.12 ± 0.042 N/kg per 0.1 m/s), while the propulsion impulse increase was similar between legs.

**Conclusions:**

PwCS showed reduced paretic leg contribution to forward propulsion across various gait speeds. The relative paretic contribution for propulsion peak remained constant while it increased with gait speed for propulsion impulse, especially in those with greater asymmetry at their comfortable walking speed. Furthermore, all participants were able to increase paretic propulsion peak and impulse above their propulsion at comfortable walking speed, suggesting some residual paretic capacity.

**Supplementary Information:**

The online version contains supplementary material available at 10.1186/s12984-025-01620-0.

## Introduction

Although most persons with chronic stroke (PwCS) regain the ability to walk independently, their gait often remains impaired [1, 2]. Gait is commonly assessed with an individual’s gait speed, which has been described as ‘the sixth vital sign’ [3]. Given its strong association with functional capacity, community ambulation and overall quality of life [4–6], increasing gait speed is a common therapy goal.

PwCS often exhibit reduced gait speed due to impaired propulsive force generation with the paretic leg [7–9]. This reduced paretic propulsion is likely due to muscle weakness [10, 11], loss of selective motor control [12], and/or balance impairments and reduced limb loading [13]. As a result, PwCS compensate by relying more on the propulsion of their non-paretic leg and increasing paretic hip pull-off to initiate the swing-phase during gait [9, 11, 14]. However, these compensatory strategies result in asymmetrical gait patterns, which are often associated with high metabolic costs [15–17].

A recent review highlighted mixed results for interventions targeting paretic propulsion [18]. Although most studies report improved gait speeds, only half of them report concurrent improvements in paretic propulsion metrics [18]. This suggests that faster gait speeds may be achieved through compensatory strategies rather than true improvement in paretic leg function [7, 18, 19]. Conversely, interventions that do show concurrent improvements of gait speed and paretic propulsion metrics appear to have in common that they target an individual’s residual paretic propulsion capacity [8, 18–22]. To correctly interpret such changes in propulsion metrics when gait speed changes concurrently, it is essential to understand how gait speed alone affects these metrics.

Paretic propulsion is typically evaluated using the anteroposterior vector of the ground reaction force (AP-GRF), adjusted for body weight. Key metrics include propulsion peak (peak AP-GRF), propulsion impulse (the integral of AP-GRF over time), and propulsion symmetry (the relative contribution of the paretic leg to total propulsion) [21]. Propulsion symmetry has been proposed as a primary propulsion metric in post-stroke gait analysis [7]. In controls, propulsion is symmetrical between both legs [23]. In addition, the propulsion peak increases with gait speed [23, 24], while the propulsion impulse remains constant across various gait speeds [24]. PwCS, however, exhibit a clear propulsion asymmetry with a larger relative contribution of the non-paretic leg at a comfortable walking speed [7, 9]. Studies assessing the effect of walking speed on propulsion symmetry by including a single walking speed above comfortable walking speed yielded contradictory results. Some studies reported no change in propulsion symmetry at a faster walking speed [20, 25], while others reported increased reliance on the non-paretic leg [8]. These discrepancies might stem from differences in gait speeds tested and because the included levels of impairment were not always clearly described. Therefore, the effect of gait speed on the modulation of propulsion in PwCS remains poorly understood. This highlights the need for systematic studies that report both relative and absolute propulsion of the paretic and non-paretic leg across a range of gait speeds in a well-defined population. Such insights are important for the design and interpretation of training or intervention studies targeting paretic propulsion.

This study aimed to investigate and characterize the relationship between incremental gait speed increases and paretic versus non-paretic propulsion in PwCS with hemiparetic gait and controls. Primary outcome was propulsion symmetry, with additional measures being the absolute propulsion peak and propulsion impulse generated by the paretic and non-paretic leg. Data from controls were used as reference values. We hypothesized an increased reliance on the non-paretic leg at higher gait speeds and therefore a decrease in propulsion symmetry at higher gait speeds. Furthermore, we expected to observe an increase in propulsion peak and impulse for both legs, with a more pronounced increase for the non-paretic leg due to compensation strategies.

## Methods

### Participants

Seventeen PwCS and sixteen healthy adults were included in this study. PwCS were included if they were > 6 months after stroke onset, had a hemiparesis secondary to a unilateral stroke and experienced self-reported gait and/or balance problems. Exclusion criteria for both groups were 1) significant self-reported pain during gait, 2) orthopaedic, neurologic, respiratory, or muscular conditions, likely to affect gait and unrelated to the consequences of a stroke, 3) inability to follow given instructions or 4) uncorrected visual impairments. An additional exclusion criterion for the PwCS was the use of a rigid ankle foot orthosis. This study was conducted in accordance with the Declaration of Helsinki and Good Clinical Practice guidelines. The Medical Research Ethics Committee of Eastern Netherlands exempted ethical approval (file number 2022–15955) as this study was not subjected to the Medical Research Involving Human Subjects Act according to Dutch Law. All subjects provided written informed consent prior to measurements.

### Apparatus

Participants walked on an instrumented split-belt treadmill within a speed-matched, virtual environment (GRAIL, Motek Medical BV, the Netherlands). Force data were collected from two embedded force plates and sampled at 1000 Hz. Kinematic data were captured by ten infrared cameras and sampled at 100 Hz (VICON, Oxford, United Kingdom). Twenty-six passive markers were placed at the following anatomical landmarks: 7 th cervical and 10 th thoracic vertebrae, suprasternal notch, xiphoid process, left and right acromion processes, humeral lateral epicondyles, ulnar styloid processes, anterior superior iliac spines, posterior superior iliac spines, lateral thighs, femoral lateral epicondyles, lateral shanks, lateral malleoli, second metatarsal heads, and calcanei.

### Experimental procedures

For each participant, self-selected comfortable walking speed was determined by gradually increasing treadmill speed until participants reported to walk at a comfortable pace. Subsequently, treadmill speed was increased and then gradually decreased until participants reported their comfortable walking speed again. The average of these two reported speeds was considered to be true comfortable walking speed. The protocol was repeated if the reported values differed more than 0.2 m/s. Participants then walked for at least 4 min at comfortable walking speed to familiarize themselves with treadmill walking. Thereafter, participants walked at a range of predetermined treadmill speeds. All gait speed conditions > 0.4 m/s lasted 2 min and ≤ 0.4 m/s lasted 3 min to ensure sufficient (> 23) strides for a reliable estimate of temporal gait parameters [26].

PwCS started with gait speed conditions ranging from 0.2 m/s up to and including comfortable walking speed in randomized order with 0.2 m/s increments. From comfortable walking speed onward, gait speed increased with 0.1 m/s for every subsequent condition until maximum gait speed was reached. Maximum gait speed was defined as the fastest treadmill speed at which a participant could fully complete the gait speed condition. Safety and feasibility of increasing treadmill speed further were discussed before each gait speed condition with the participant, researcher, and therapist. Furthermore, if exertion levels exceeded a fatigue level of thirteen on the Borg scale [27], participants were asked to rest and continue when the score dropped below 10.

Controls completed eight gait speed conditions in which treadmill speed was randomized from 0.4 to 1.6 m/s with 0.2 m/s increments and an additional condition at comfortable walking speed. All participants wore a non-weight-bearing safety harness during the measurements.

### Data analysis

Motion capture and force plate data were processed and analysed using custom software in Python (v3.11) [28]. This software, together with example data, was made publicly available on GitHub [29]. The first ten and final five seconds of each gait speed condition were excluded from analysis to ensure steady-state gait by accounting for the start and stop phase, respectively. Marker data and force plate data were filtered with a second order zero-phase low-pass Butterworth filter with a cut-off frequency of 15 Hz. Force plate data were resampled at 100 Hz to match the motion capture sampling frequency.

Propulsion was derived from the anteroposterior component of the ground reaction force. First, The stance phase was estimated using marker data of the feet [30]. Subsequently, the peak propulsion was defined by the local maximum (peak) per stance phase. Finally, the start and end of the propulsion curves were defined as the zero-crossings before and after the peak, respectively. For the detailed calculations, we refer to the provided open source Python scripts [29]. Individual steps were excluded from the analysis if both feet were (partially) placed on the same force plate to ensure a reliable propulsion estimation. Participants were excluded from the analysis if fewer than 23 steps per leg [26] were available in two or more gait speed conditions.

#### Outcome measures

**Fig. 1:**
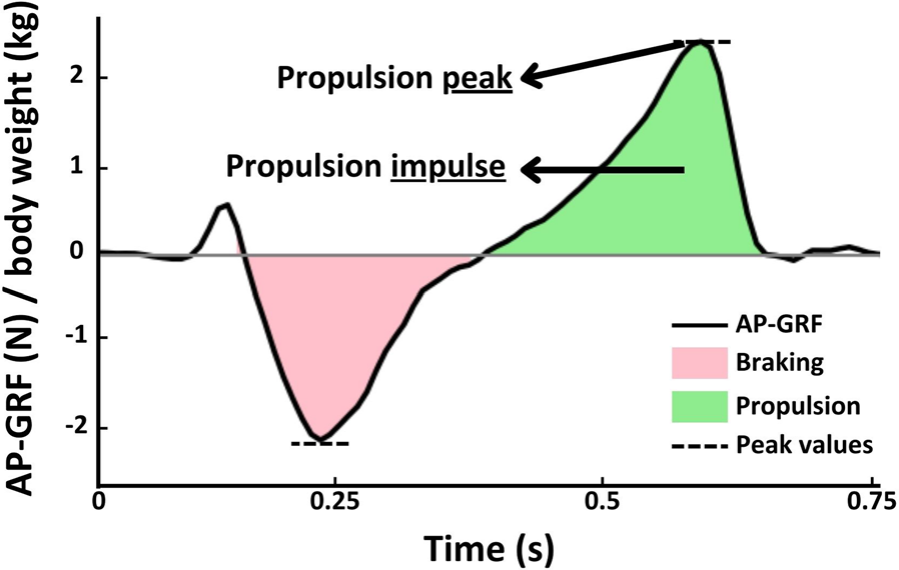
A typical example of an anteroposterior ground reaction force (AP-GRF) curve, corrected for body weight, of a control walking at a comfortable speed. The pink shaded area represents the braking impulse, green shaded area represents the propulsion impulse and dashed lines indicate the minimum and maximum values within a step

Outcome measures were computed per stance phase and averaged over all stance phases per gait speed condition per participant. Propulsion was corrected for body weight and calculated as the maximal propulsion value (N/kg), referred to as *propulsion peak*, and the area under the curve (N/kg*s), referred to as *propulsion impulse* (Fig. 1). Examples of typical paretic and non-paretic propulsion curves at various gait speeds and several atypical examples are provided in Additional file 1.

Propulsion symmetry was the primary outcome and reflects the relative contribution of the paretic leg to total propulsion. Propulsion symmetry was calculated as:

1 $$Propulsion \,symmetry= \frac{{Propulsion}_{paretic \,leg}}{{Propulsion}_{paretic \,leg}+{Propulsion}_{non-paretic\, leg}}*100\%$$

and was computed for both the propulsion peak and propulsion impulse. A symmetry of 50% indicates equal contribution of the paretic and non-paretic leg and values < 50% indicate a lower contribution of the paretic leg. For controls, propulsion symmetry was defined as the relative contribution of the left leg to the total propulsion. The absolute values of propulsion peak and propulsion impulse of both the paretic and non-paretic leg were used for secondary analysis.

The level of functional impairment was assessed for each PwCS using the following clinical scores: MiniBEST, Functional Ambulation Category (FAC), Timed Up and Go (TUG) and Activities-specific Balance Confidence (ABC) scale.

#### Statistical analysis

Participant characteristics were presented as mean and standard deviation for continuous variables for normally distributed data or as median and interquartile range for non-normally distributed data. Dichotomous variables were presented as ratios. Between group-differences were analysed using an unpaired t-test for normally distributed data and a Mann–Whitney U-test for non-normally distributed data.

To address the primary aim, two Linear Mixed Models (LMMs) were used to study the relation between gait speed and *propulsion peak symmetry* and *propulsion impulse symmetry* (Eq. 2):

2 $$Propulsion \,symmetry\left(ij\right)=\beta 0+\beta 1\left(Gait \,speed\left(ij\right)\right)+\beta 2\left(Symmetry \,at \,CWS\left(ij\right)\right)+\beta 3\left(Gait\, speed\left(ij\right)\times\, Symmetry\, at \,CWS\left(ij\right)\right) +b0\left(i\right)+b1\left(i\right)\left(Gait \,speed\left(ij\right)\right)+\epsilon \left(ij\right)$$

for observation ‘*j*’ in participant ‘*i*’. Gait speed was the fixed effect ($\beta$) of interest in both models and provided a group-estimate to answer the primary research question. Propulsion symmetry at comfortable walking speed was included as an interaction term to examine whether the relationship between gait speed and propulsion symmetry varied depending on the level of propulsion symmetry at one’s comfortable walking speed. The fixed effect of propulsion symmetry at comfortable walking speed is not meaningful and was therefore not reported. Random effects (*b*) included intercepts and slopes, accounting for between-subject variability and the interdependence of repeated measures within participants.

To address the secondary aim, two additional LMMs were used to study the relation between gait speed and *propulsion peak* and *propulsion impulse* in the paretic and non-paretic leg (Eq. 3):

3 $$Propulsion(ij)=\beta 0+\beta 1Gait\, speed(ij)+\beta 2Leg(ij)+\beta 3(Gait\, speed(ij)\times Leg(ij))+b0(i)+b1(i)Gait\, speed(ij)+b2(i)Leg(ij)+b3(i)(Gait\, speed(ij)\times Leg(ij))+\epsilon (ij)$$

for observation ‘*j*’ in participant ‘*i’*. For both these models, fixed effects were gait speed and leg (paretic versus non-paretic). If no interaction between gait speed and leg was found, the gait speed estimate ($\beta$) was used to answer the secondary research question. If an interaction was found, the gait speed estimates of two separate models per leg were used to answer the secondary research question. Random effects (*b*) were participant (intercept) and slope * leg to allow for between-subject and between-leg variability of the effect. All models were fitted in R version 4.3.3 using the lmer function in the lme4 package.

The model fits were evaluated on normality, homoscedasticity and non-linearity using the following diagnostic plots: Q-Q plot, a histogram of residuals and residuals plotted versus fitted values. The Kenward-Roger method was used to adjust the degrees of freedom and correct the fixed effects estimates. Results were deemed significant at the α = 0.05 level.

## Results

### Participants

Participant characteristics are presented in Table 1. Of the seventeen PwCS, two were excluded from the analysis due to insufficient steps to analyse in six out of eight, and six out of six gait speed conditions. Of the fifteen PwCS included in the analysis, two had a single gait speed condition (0.6 m/s and 1.4 m/s) excluded due to an insufficient number of steps.

**Table 1:** Overview of participant characteristics

	Persons with chronic stroke	Controls	p-value
N	15	16	
Male/Female	10/5	7/9	0.20
Age (years)	67 ± 11	23 ± 3	** < 0.001**
Weight (kg)	80 [75, 90]	68 [63, 89]	** < 0.001**
Time post-stroke (months)	23 [16, 48]		
Functional Ambulation Category (1–5)	4 [3, 5]		
MiniBEST score (0–28)	17 ± 3		
N participants with MiniBest score < 19	11		
Timed Up and Go (s)	14.4 ± 3.7		
Activities-Specific Balance Confidence Scale (0–100%)	66 ± 21		

Values are presented as mean ± SD or median [IQR]. Between-group comparisons are independent t-tests if normally distributed or Mann–Whitney U tests if non-normally distributed. Male – female ratios between groups were tested with a Chi-square test. Bold p-values indicate a statistically significant difference between persons with chronic stroke and controls

### Gait speed and propulsion symmetry

**Fig. 2:**
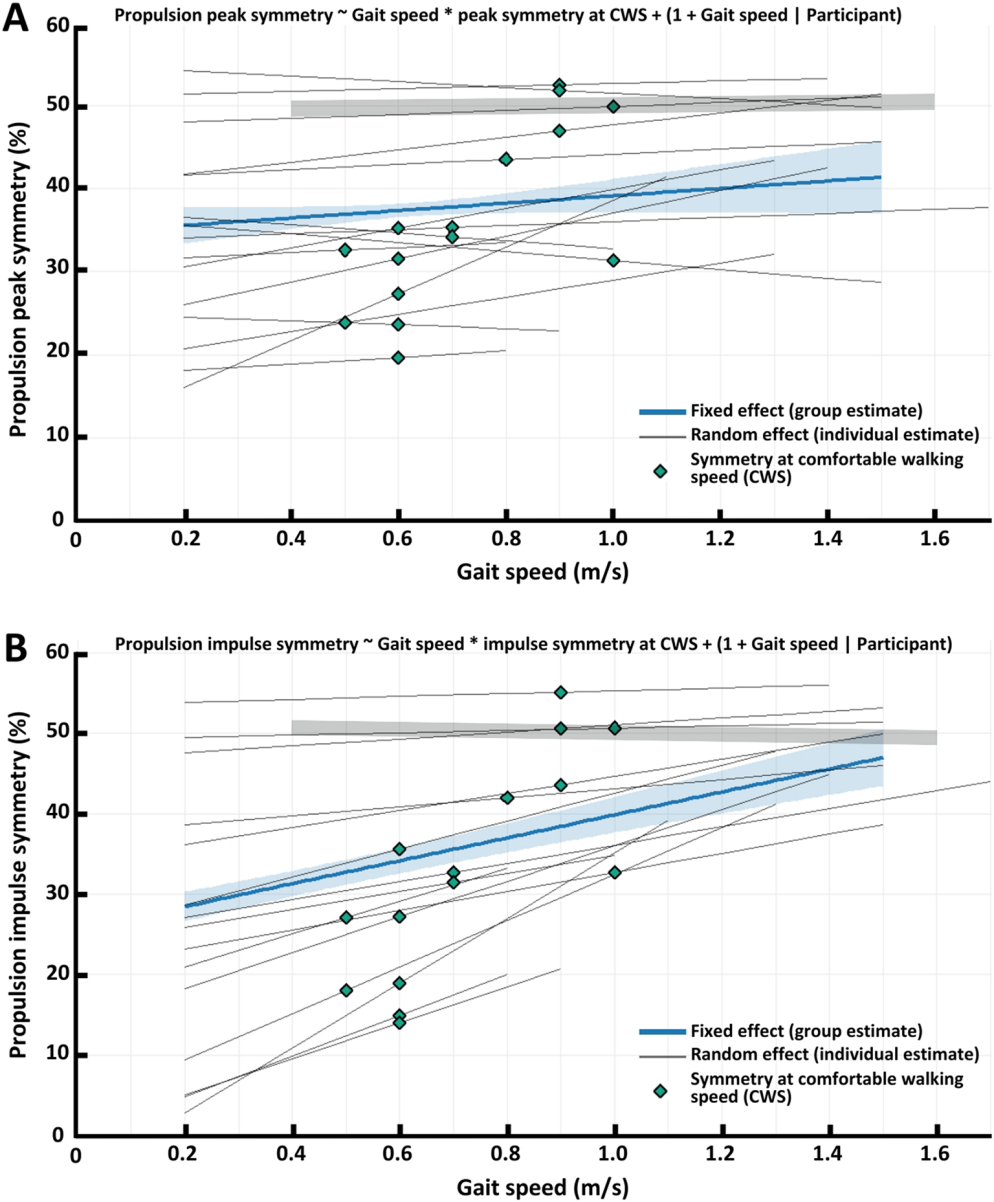
Linear mixed models describing the relation between gait speed and propulsion peak (**A**) and propulsion impulse (**B**) symmetry in persons with chronic stroke. A value of 50 represents perfect symmetry between the paretic and non-paretic leg. Blue, thick lines represent the fixed effect of gait speed (group-estimate). Black, thin lines represent random effects (individual estimates). Diamonds represent the estimated symmetry at an individual’s comfortable walking speed (CWS). Blue and grey shaded area’s represent 95% confidence intervals of the fixed effect for persons with chronic stroke and controls, respectively

The two LMMs to study the relationship between gait speed and *propulsion peak symmetry* and *propulsion impulse symmetry* in PwCS, including model specifications, are presented in Fig. 2A and 2B, respectively. Two additional LMMs were used to describe this relationship in controls. All models fitted the data well, as indicated by low residual, and scaled variances (Additional file 2). Diagnostic plots revealed no substantial deviations from normality, homoscedasticity, or linearity.

#### Propulsion peak symmetry

In PwCS, no significant fixed effect of gait speed on propulsion peak symmetry was observed (β = 0.12, SE = 0.090, t = 1.4_(12.9)_, p = 0.19) and there was no significant interaction between gait speed and propulsion peak symmetry at comfortable walking speed (β = −0.24, SE = 0.024, t = −0.94_(12.2)_, p = 0.36). Random effects showed considerable variability of individual slopes across participants (SD = 0.093).

In controls, gait speed showed no significant fixed effect on propulsion peak symmetry (β = 0.0052, SE = 0.0088, t = 0.60_(15)_, p = 0.56) and propulsion peak symmetry was centred around 0.5 as illustrated by the intercept of 0.50 (SE = 0.012). Random effects show relatively low slope variability between participants (SD = 0.033).

#### Propulsion impulse symmetry

In PwCS, gait speed had a consistent positive fixed effect (improved symmetry) on propulsion impulse symmetry (β = 0.39, SE = 0.048, t = 8.2_(14.4)_, p < 0.001). This effect was stronger (larger slope) for participants with lower propulsion symmetry at comfortable walking speed, as indicated by the significant interaction (β = −0.715, SE = 0.13, t = −5.6_(12.6)_, p < 0.001). Substantial inter-individual differences were further illustrated by the slope variability (random effect) between participants (SD = 0.057).

In controls, no significant effect of gait speed on propulsion impulse symmetry was observed (β = −0.0043, SE = 0.0080, t = −0.54_(15)_, p = 0.60) and propulsion impulse symmetry was centred around 0.5 as illustrated by the intercept of 0.51 (SE = 0.012). Random effects show relatively low variability between participants (SD = 0.019).

### Gait speed and paretic and non-paretic propulsion

**Fig. 3:**
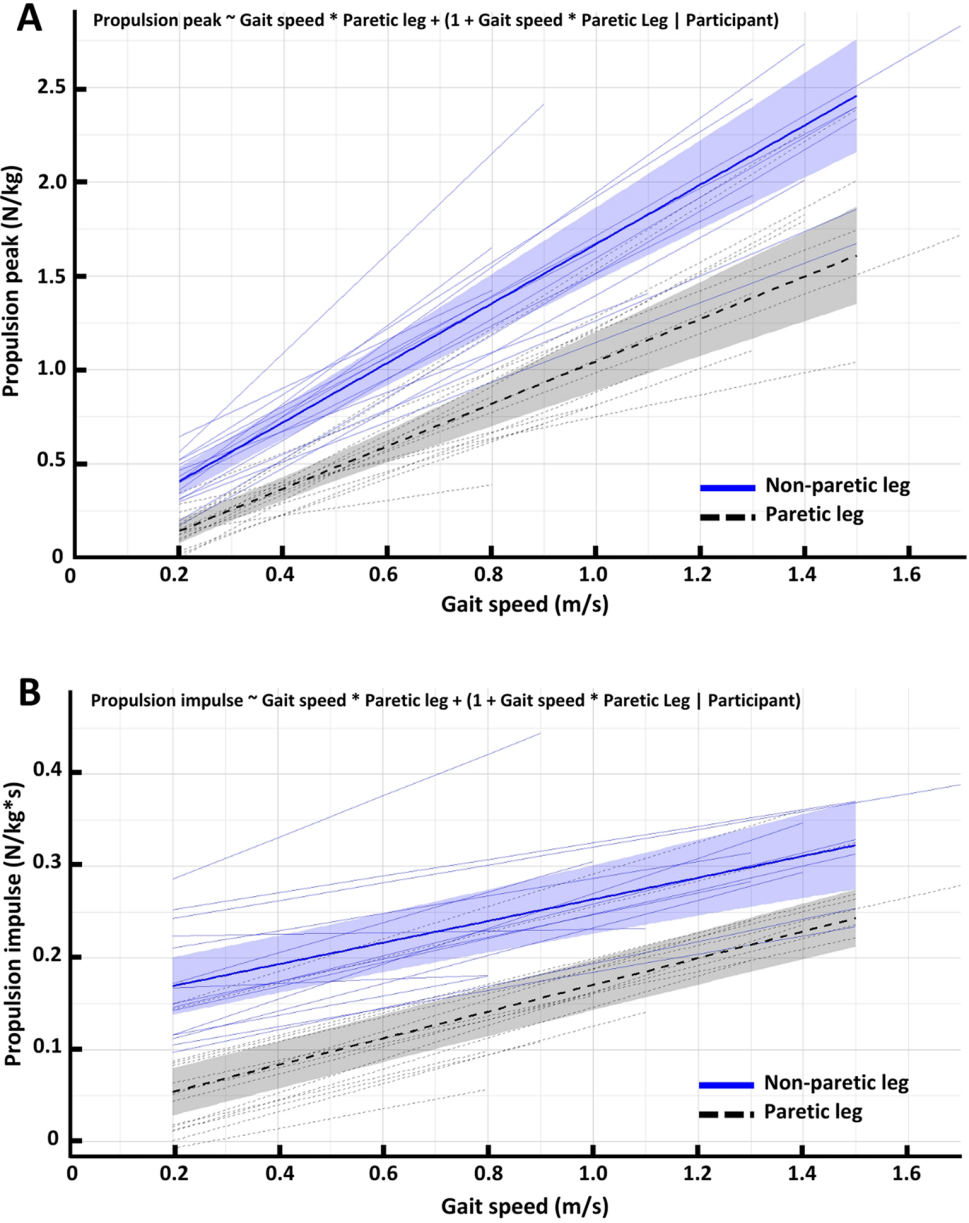
Linear mixed models describing the relation between gait speed and propulsion peak (**A**) and propulsion impulse (**B**) in the paretic and non-paretic leg of PwCS. Blue, solid lines represent the non-paretic leg’s fixed effect (thick line) and random effects (thin lines). Black, dashed lines represent the paretic leg’s fixed effect (thick line) and random effects (thin lines). Shaded areas represent 95% confidence intervals of the group-estimates (fixed effect)

The LMMs used to study the relationship between gait speed and paretic and non-paretic *propulsion peak* and *propulsion impulse*, including model specifications, are presented in Fig. 3A and B, respectively. Two additional LMMs described this relationship in controls. All models fitted the data well, as indicated by low residual, and scaled variances (Additional file 3). Diagnostic plots revealed no substantial deviations from normality, homoscedasticity, or linearity.

#### Propulsion peak

In PwCS, a significant interaction between the effect of gait speed and leg was observed (β = −0.47, SE = 0.16, SD = 0.57, t = −2.6_(13.0)_, p = 0.013), indicating different propulsion peak increases between the paretic and non-paretic leg with increasing gait speed. Post-hoc analysis of the effect of gait speed per leg showed that non-paretic propulsion peak increased with 0.16 ± 0.043 N/kg per 0.1 m/s (β = 1.6, SE = 0.12, SD = 0.043, t = 13_(13.0)_, p < 0.001), whereas paretic propulsion peak increased with 0.12 ± 0.042 N/kg per 0.1 m/s (β = 1.2, SE = 0.12, SD = 0.042, t = 9.69_(12.9)_, p < 0.001). Additionally, a significant, negative, fixed effect of the paretic leg (β = −0.17, SE = 0.054, SD = 0.17, t = −3.1_(12.9)_, p = 0.0090) was found, indicating an overall reduction of propulsion peak by the paretic leg compared to the non-paretic leg. This overall reduction of propulsion peak by the paretic leg showed substantial variability between participants, as illustrated by the large standard deviation (SD = 0.17). In controls, a strong, positive fixed effect of gait speed on propulsion peak was observed with little inter-individual differences (β = 1.73, SE = 0.041, SD = 0.15, t = 42_(15)_, p < 0.001).

#### Propulsion impulse

In PwCS, no significant interaction between the effect of gait speed and leg was observed (β = 0.026, SE = 0.022, SD = 0.071, t = 1.2_(11.8)_, p = 0.25), meaning that both legs showed similar effects of gait speed on propulsion impulse. There was an overall strong, positive effect of gait speed (β = 0.12, SE = 0.021, SD = 0.070, t = 5.8_(12.0)_, p < 0.001), with an average increase of 0.012 ± 0.0070 N/kg*s per 0.1 m/s for both legs. The paretic leg showed an overall average reduction of 0.12 ± 0.094 N/kg*s compared to the non-paretic leg, as indicated by the significant, negative, fixed effect of the paretic versus non-paretic leg (β = −0.12, SE = 0.026, SD = 0.094, t = −4.8_(12.8)_, p < 0.001). This negative effect of paretic versus non-paretic leg differed substantially between participants, as illustrated by the large standard deviation (SD = 0.094).

In controls, a consistent yet moderately strong positive fixed effect of gait speed on propulsion impulse was observed (β = 0.0083, SE = 0.0076, SD = 0. 0026, t = 11_(15)_, p < 0.001).

## Discussion

The current study examined the inter-limb modulation of propulsion in persons with chronic stroke (PwCS) and controls while walking at various gait speeds. Propulsion was characterized by means of the two most used propulsion metrics: propulsion *peak* and propulsion *impulse.* PwCS presented a propulsion peak and propulsion impulse asymmetry at all gait speeds, whereas controls maintained symmetrical propulsion peak and impulse at all gait speeds. In PwCS included in this study, propulsion *peak* symmetry was not altered by gait speed. However, propulsion *impulse* symmetry improved with increasing gait speed, particularly in PwCS who had higher propulsion asymmetry at their comfortable walking speed.

### Propulsion peak

While controls showed symmetrical peak propulsion, most PwCS presented clear asymmetry across all gait speed conditions (Fig. 2A), consistent with the literature [7, 9, 17, 18]. Propulsion peak symmetry did not change across a range of gait speeds in PwCS. This finding aligns with earlier studies that found no significant change in propulsion peak symmetry at a single faster walking speed compared to a comfortable walking speed [20, 31]. Hence, the relative contribution of the paretic leg to propulsion peak generation seems to remain constant despite varying propulsion demand on both legs. However, we observed large inter-individual variability. Some participants improved symmetry, while others decreased symmetry with increasing gait speeds, mirroring previous findings [31].

To further understand the changes in propulsion symmetry, our secondary analysis described changes in propulsion peak for each leg across all gait speed conditions. In controls, both legs showed a consistent increase in propulsion peak with increasing gait speed. In PwCS, the paretic leg generated a lower propulsion peak in all gait speed conditions compared to the non-paretic leg. Nonetheless, each PwCS was able to modulate their non-paretic and paretic propulsion peak to increasing gait speeds, even above comfortable walking speed. This increase was slightly larger for the non-paretic leg (0.16 ± 0.043 N/kg per 0.1 m/s) compared to the paretic leg (0.12 ± 0.042 N/kg per 0.1 m/s). These findings align with previous work [8, 20] that demonstrated a voluntary increase in paretic peak propulsion at gait speeds above comfortable walking speed. This further supports the growing notion that PwCS can generate more propulsion with their paretic leg compared to their propulsion at a comfortable walking speed [17–19, 32, 33].

### Propulsion impulse

Previous research has shown propulsion impulse asymmetry at preferred walking speed in PwCS [7, 9, 17, 18]. Similarly, most PwCS in the current study exhibited a clear asymmetry in their propulsion impulse across all gait speeds (Fig. 2B). However, contrary to our hypothesis, propulsion impulse symmetry improved with increasing gait speeds, especially in participants with greater propulsion asymmetry at their comfortable walking speed and to a lesser extend in those closer to symmetry (50%) at this speed. This finding contrasts with a previous study that found no effect of gait speed on propulsion impulse symmetry when comparing preferred speed to the fastest comfortable speed [25]. The discrepancy may relate to differences in functional impairment and gait speeds between studies. In their study, the average comfortable walking speed, which reflects functional impairment [34], was 0.45 ± 0.25 m/s versus 0.71 ± 0.18 m/s in the current study. In addition, their comparison to a single faster gait speed (0.69 ± 0.38 m/s) may have been less sensitive to reveal changes in symmetry than our wider range of speeds.

Our secondary analysis revealed that the paretic and non-paretic leg’s propulsion impulse increased at the same rate with increasing gait speed. However, the paretic leg’s propulsion impulse was lower in all gait speed conditions compared to the non-paretic leg. Controls also significantly modulated propulsion impulse with gait speed, which contradicts a previous study that reported no difference in propulsion impulse between a ‘slow’, ‘self-selected’, and ‘fast’ condition in controls [24]. This discrepancy likely stems from methodological differences. Deffeyes and colleagues reported large between-subject and within-subject variability in the range of gait speeds, which they believe may have masked subtle gait speed effects [24].

### Propulsion peak versus propulsion impulse

Propulsion impulse symmetry increased but propulsion peak symmetry did not, while there is a significant interaction for leg in propulsion peak but not in propulsion impulse. Although these findings may seem contradictory, they can be understood within the context of the propulsion symmetry equation (Eq. 1). For propulsion peak, there is a larger *absolute* increase of the non-paretic leg compared to the paretic leg, but the *relative* (percentage) increase is similar between legs due to the overall lower paretic propulsion peak across gait speeds. This led to similar relative increases of the denominator (paretic propulsion) and numerator (paretic propulsion + non-paretic propulsion) of the equation (Eq. 1), resulting in no change in propulsion peak symmetry. In contrast, for propulsion impulse, the *absolute* increase was similar in both legs, but the paretic leg has a larger *relative* (percentage) increase due to a lower overall propulsion impulse across all speeds. As a result, the relative contribution of the paretic leg increases and propulsion impulse symmetry improved*.*.

The finding that the absolute increase of propulsion peak is larger for the non-paretic leg compared to the paretic leg, while propulsion impulse increases similarly between legs may reflect the distinct concepts of propulsion they represent. Propulsion peak is a single value indicating the maximum propulsive force, while propulsion impulse includes the temporal aspect of the push off [9, 21]. As previously found, increasing gait speed can lead to a more symmetrical gait pattern after a stroke, shown by improved support time, hip extension, knee flexion, and step length of the paretic side [35]. These changes contribute to the generation of paretic propulsion for a relatively prolonged time within a stride [36], which might therefore be reflected in a relatively wider and larger propulsion impulse but not necessarily a higher propulsion peak. This may explain why the paretic leg is able to produce a similar propulsion impulse increase, but not propulsion peak increase, compared to the non-paretic leg.

### Implications

Our findings have several clinical and scientific implications. However, we do not intend to provide advice on what propulsion metric is ‘best’ to use, but rather highlight the distinct concepts of propulsion they represent. What metric to use in future work should primarily depend on the study’s purpose. Propulsion peak might for example be of particular interest when peak forces are important, such as in knee osteoarthritis [37]. On the other hand, propulsion impulse might be of interest when an individual’s overall propulsion generation is examined, as it also accounts for the temporal aspect of propulsion generation [21].

Nonetheless, our findings emphasize the importance to consider the relationship between gait speed and propulsion (symmetry) in training and intervention studies. Given that most intervention studies report positive effects on gait speed [18], it is crucial to identify whether improvements in propulsion are due to the intervention itself or simply the result of walking at higher speeds. We recommend reporting both propulsion symmetry and absolute propulsion metrics, as symmetry alone does not fully capture underlying physiological changes in propulsion. Furthermore, the group-estimates presented in this study may be used as a rough correction for the effect of gait speed at the group-level. However, these group-level estimates should not be applied at an individual level due to the substantial variability between subjects, which appears to depend on one’s baseline level of propulsion symmetry at comfortable walking speed. Therefore, future studies targeting paretic propulsion should consider performing pre- and post-measurements at a single fixed gait speed or several fixed speeds to isolate propulsion improvements from concurrent speed effects.

Our data also showed that PwCS can modulate paretic propulsion to meet the demands of increased gait speed beyond a comfortable pace. This raises the question of why PwCS do not fully utilize this residual capacity at their comfortable walking speed [7, 19, 23, 38]. Previous work showed that PwCS seek a balance between enhancing paretic propulsion, managing energetic costs [16] and maintaining balance control [13, 39]. In the present study, increasing gait speed seems to be a simple straightforward method to activate the residual capacity of the paretic leg. Importantly, this applies not only to maximum gait speeds, which are not always feasible for safety reasons or fatigue, but also to more marginal increases.

### Study limitations

There are some limitations to consider when interpreting the results of this study. PwCS presented with a large heterogeneity in levels of propulsion symmetry and the current sample size was insufficient to categorize functional subgroups based on the levels of propulsion symmetry. In addition, the results presented in this study are based on participants with a hemiparesis, substantial functional impairment and who can walk independently at a comfortable walking speed above 0.5 m/s. These results should not be generalized to PwCS outside these criteria such as household ambulators (< 0.4 m/s), who are generally more severely impaired [40]. Finally, the controls in this study were significantly younger compared to the PwCS. Although the control data served primarily as a reference for individuals without gait asymmetry, the age difference may limit the interpretation of direct comparisons due to unaccounted age effects (41).

## Conclusion

PwCS presented pronounced propulsion peak and propulsion impulse asymmetry across various gait speeds. Increasing gait speed did not affect propulsion peak symmetry, although significant inter-individual variability was observed. In contrast, propulsion impulse symmetry improved with increasing gait speeds, particularly for those with greater asymmetry at their comfortable walking speed. PwCS were able to modulate their absolute propulsion peak and impulse of the paretic and non-paretic leg to increasing gait speeds beyond comfortable walking speed, suggesting some residual paretic capacity.

## Supplementary Information

Additional file 1: Title of data: Paretic and non-paretic propulsion curves at various gait speeds, including atypical examples. Description of data: Anteroposterior ground reaction forcecurves of A) a typical example of the paretic and non-paretic leg at various gait speeds and corresponding propulsion peak and impulse symmetries, B) an atypical example of a negative peak within the propulsion impulse which is considered to oppose propulsion and subtracted from the propulsion impulse, and C) an example of a negative peak after the propulsion impulse which is considered to be unrelated to the propulsion impulseand therefore not taken into account. *IC = initial contact; TC = terminal contact.*

Additional file 2: Title of data: Linear mixed model results for propulsion peak and impulse symmetry. Description of data: Results of the linear mixed model describing the relationship between gait speed and propulsion peak symmetry and propulsion impulse symmetry in persons with chronic stroke

Additional file 3: Title of data: Linear mixed model results for absolute propulsion peak and impulse per leg. Description of data: Results of the linear mixed model describing the relationship between gait speed and paretic and non-paretic propulsion peak and propulsion impulse

## Data Availability

No datasets were generated or analysed during the current study.

## Abbreviations

AP-GRF: Anteroposterior ground reaction force
CWS: Comfortable walking speed
IQR: Interquartile range
LMM: Linear mixed model
PwCS: Persons with chronic stroke
SD: Standard deviation
SE: Standard error
