# Supplementary figures and images for: Gait speed-dependent modulation of paretic versus non-paretic propulsion in persons with chronic stroke
Source: J Neuroeng Rehabil. 2025 May 8;22:108. doi: 10.1186/s12984-025-01620-0 (PMC12063273; doi:10.1186/s12984-025-01620-0)

Additional file 1

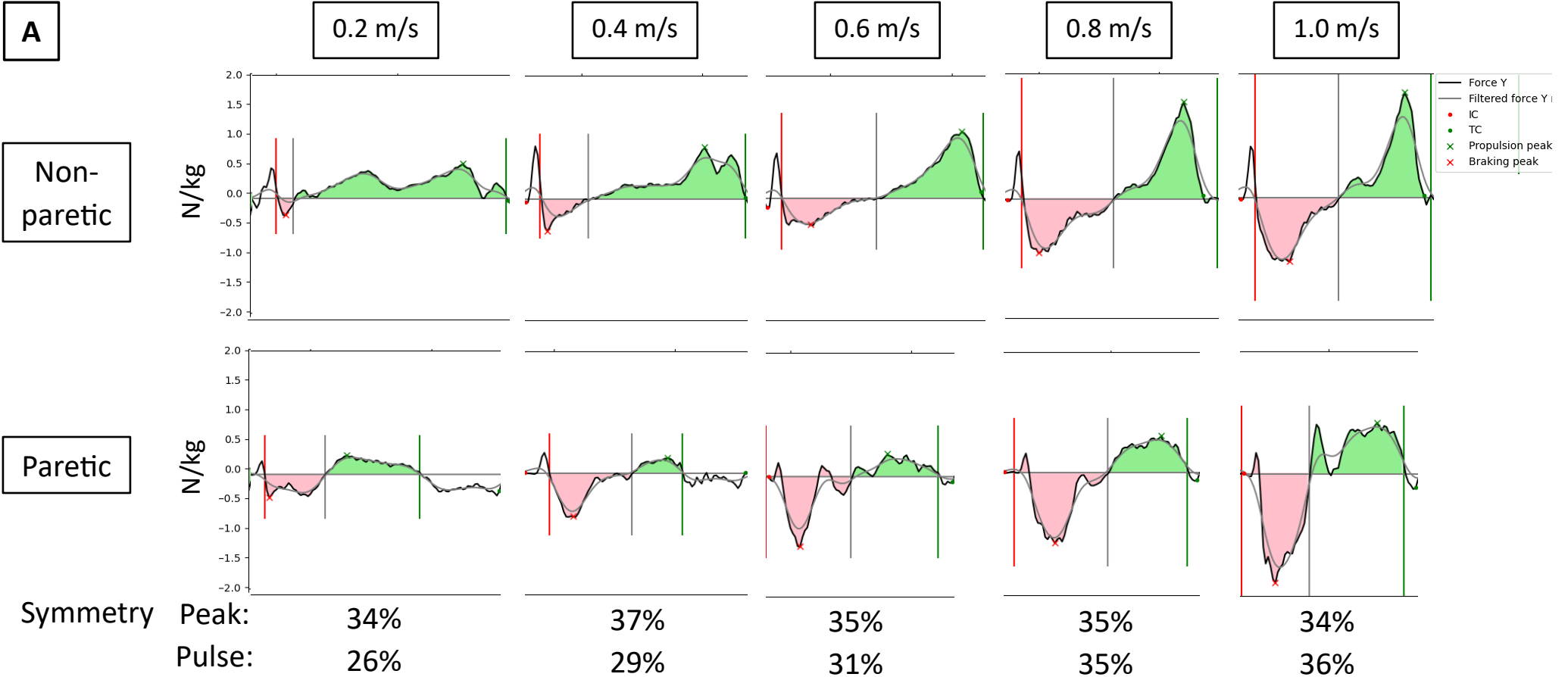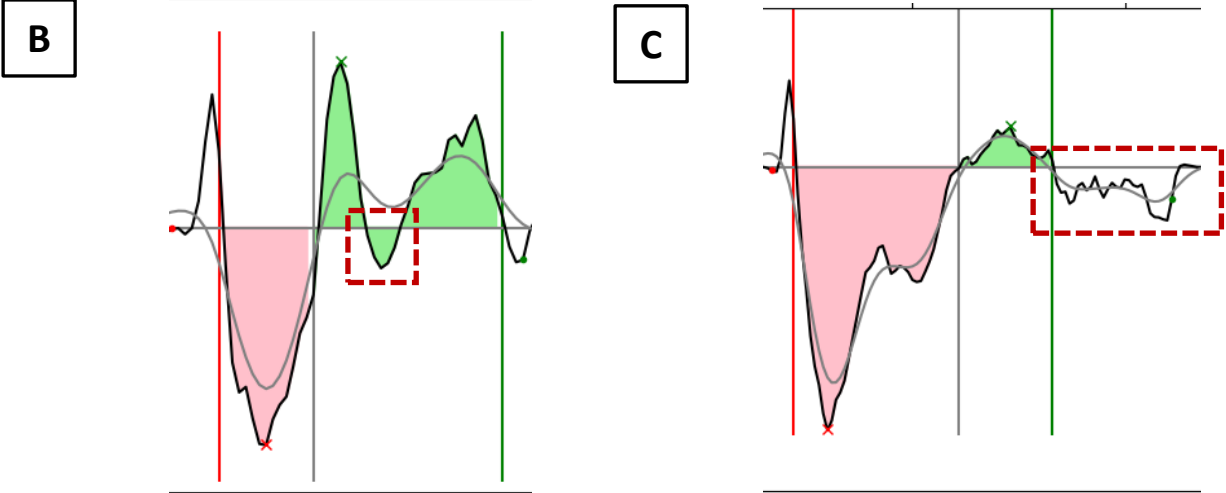

Supplement: Supplementary file 1 — Additional file 1: Title of data: Paretic and non-paretic propulsion curves at various gait speeds, including atypical examples. Description of data: Anteroposterior ground reaction forcecurves of A) a typical example of the paretic and non-paretic leg at various gait speeds and corresponding propulsion peak and impulse symmetries, B) an atypical example of a negative peak within the propulsion impulse which is considered to oppose propulsion and subtracted from the propulsion impulse, and C) an example of a negative peak after the propulsion impulse which is considered to be unrelated to the propulsion impulseand therefore not taken into account. IC = initial contact; TC = terminal contact. [file 12984_2025_1620_MOESM1_ESM.pdf]
