# Supplementary material for: Gait speed-dependent modulation of paretic versus non-paretic propulsion in persons with chronic stroke
Source: J Neuroeng Rehabil. 2025 May 8;22:108. doi: 10.1186/s12984-025-01620-0 (PMC12063273; doi:10.1186/s12984-025-01620-0)
Supplement: Supplementary file 2 — Additional file 2: Title of data: Linear mixed model results for propulsion peak and impulse symmetry. Description of data: Results of the linear mixed model describing the relationship between gait speed and propulsion peak symmetry and propulsion impulse symmetry in persons with chronic stroke [file 12984_2025_1620_MOESM2_ESM.pdf]

Additional file 2: Table S1 Results of the linear mixed model describing the relationship between gait speed and *propulsion peak symmetry* and *propulsion pulse symmetry* in persons with chronic stroke.

		**Fixed effects**				
		Estimate (β)	Std. Error	df	t-value	p-value
**Propulsion pulse symmetry**	(Intercept)	**-0.21**	**0.033**	**13.5**	**-6.3**	**< .001**
	Gait speed	**0.39**	**0.048**	**14.4**	**8.20**	**< .001**
	Gait speed * propulsion symmetry at comfortable walking speed	**-0.72**	**0.13**	**12.6**	**-5.6**	**< . 001**
**Propulsion peak symmetry**	(Intercept)	-0.038	0.057	12.6	-0.67	0.51
	Gait speed	0.12	-0.057	12.9	1.4	0.20
	Gait speed * propulsion symmetry at comfortable walking speed	-0.22	0.24	12.2	-0.93	0.37

		**Random effects**		
		Variance	SD	Correlation
**Propulsion pulse symmetry**	(Intercept)	0.0015	0.039	
	Slope	0.0033	0.057	- 0.68
**Propulsion peak symmetry**	(Intercept)	0.0034	0.058	
	Slope	0.0086	0.093	-0.98

	**Model fit**		
	Residual variance (σ² (SD))	Scaled Residuals (median [1Q, 3Q])	Scaled residuals (min, max)
**Propulsion pulse symmetry**	0.00081	0.029 [-0.44, 0.50]	-3.2, 2.2
**Propulsion peak symmetry**	0.00073	0.043 [-0.47, 0.55]	-2.6, 2.5

Bold values represent significant effects
