# Supplementary material for: Gait speed-dependent modulation of paretic versus non-paretic propulsion in persons with chronic stroke
Source: J Neuroeng Rehabil. 2025 May 8;22:108. doi: 10.1186/s12984-025-01620-0 (PMC12063273; doi:10.1186/s12984-025-01620-0)
Supplement: Supplementary file 3 — Additional file 3: Title of data: Linear mixed model results for absolute propulsion peak and impulse per leg. Description of data: Results of the linear mixed model describing the relationship between gait speed and paretic and non-paretic propulsion peak and propulsion impulse [file 12984_2025_1620_MOESM3_ESM.pdf]

Additional file 3: Table S2 Results of the linear mixed model describing the relationship between gait speed and paretic and non-paretic *propulsion peak* and *propulsion pulse.*

		**Fixed effects**				
		Estimate (β)	SE	df	t-value	p-value
**Propulsion pulse**	(Intercept)	**0.15**	**0.018**	**12.4**	**8.2**	**< .001**
	Gait speed	**0.12**	**0.021**	**12.0**	**5.8**	**< .001**
	Leg#	**-0.12**	**0.026**	**12.8**	**-4.8**	**< .001**
	Gait speed * Leg#	0.026	0.022	11.8	1.21	0.25
**Propulsion peak**	(Intercept)	0.10	0.050	12.9	2.0	0.064
	Gait speed	**1.55**	**0.12**	**13.0**	**12**	**< .001**
	Leg#	**-0.18**	**0.057**	**12.9**	**-3.1**	**0.0095**
	Gait speed * Leg#	**-0.43**	**0.17**	**13.0**	**-2.57**	**0.025**
		**Random effects**				
		Variance	SD	Correlation		
**Propulsion pulse**	(Intercept)	0.0042	0.064			
	Slope	0.0049	0.070	-0.30		
	Leg#	0.0088	0.094	-0.88	0.32	
	Slope * Leg#	0.0051	0.071	0.13	-0.95	-0.19
**Propulsion peak**	(Intercept)	0.025	0.16			
	Slope	0.18	0.43	-0.40		
	Leg#	0.029	0.17	-0.47	0.17	
	Slope * Leg#	0.35	0.59	-0.34	-0.68	-0.08

	**Model fit**		
	Residual variance (σ^2 (SD))	Scaled Residuals (median [1Q, 3Q])	Scaled residuals (min, max)
**Propulsion pulse**	0.00028 (0.017)	-0.012 [-0.56, 0.56]	-2.1, 2.5
**Propulsion peak**	0.0092 (0.096)	0.022 [-0.57, 0.55]	-4.7, 2.4

# Paretic leg as compared to non-paretic leg; Bold values represent significant effects
